# Supplementary material for: Fungicidal Activity of Recombinant Javanicin against Cryptococcus neoformans Is Associated with Intracellular Target(s) Involved in Carbohydrate and Energy Metabolic Processes
Source: Molecules. 2021 Nov 20;26(22):7011. doi: 10.3390/molecules26227011 (PMC8618071; doi:10.3390/molecules26227011)
Supplement: Supplementary file 1 [file molecules-26-07011-s001.zip › molecules-1455111-supplementary.pdf]

Supporting Information

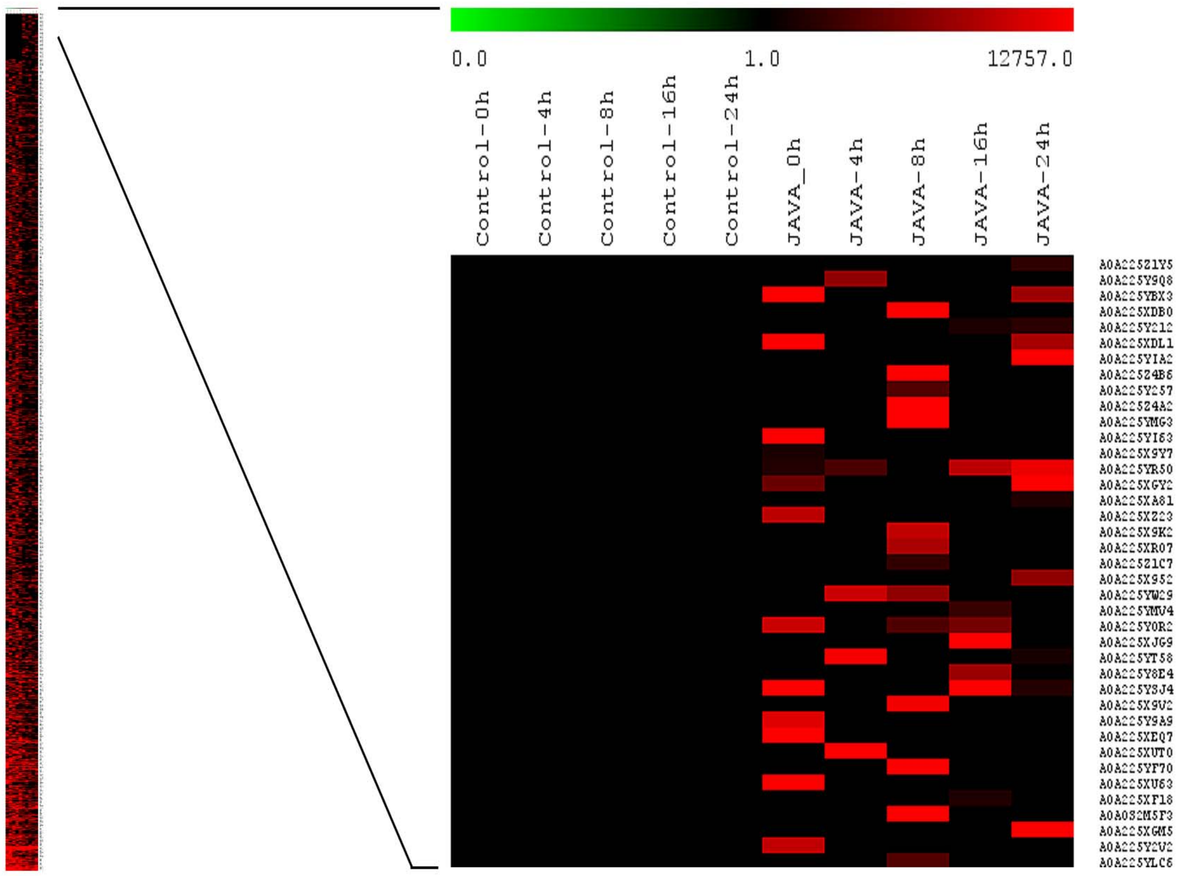

Supplementary Figure S1. Heat map of fungal protein hits in various time points between untreated control and r-javanicin treated group with a significantly difference (p <0.05). The x-axis is untreated control and peptide treated at various time point and the y-axis is the proteins identified.

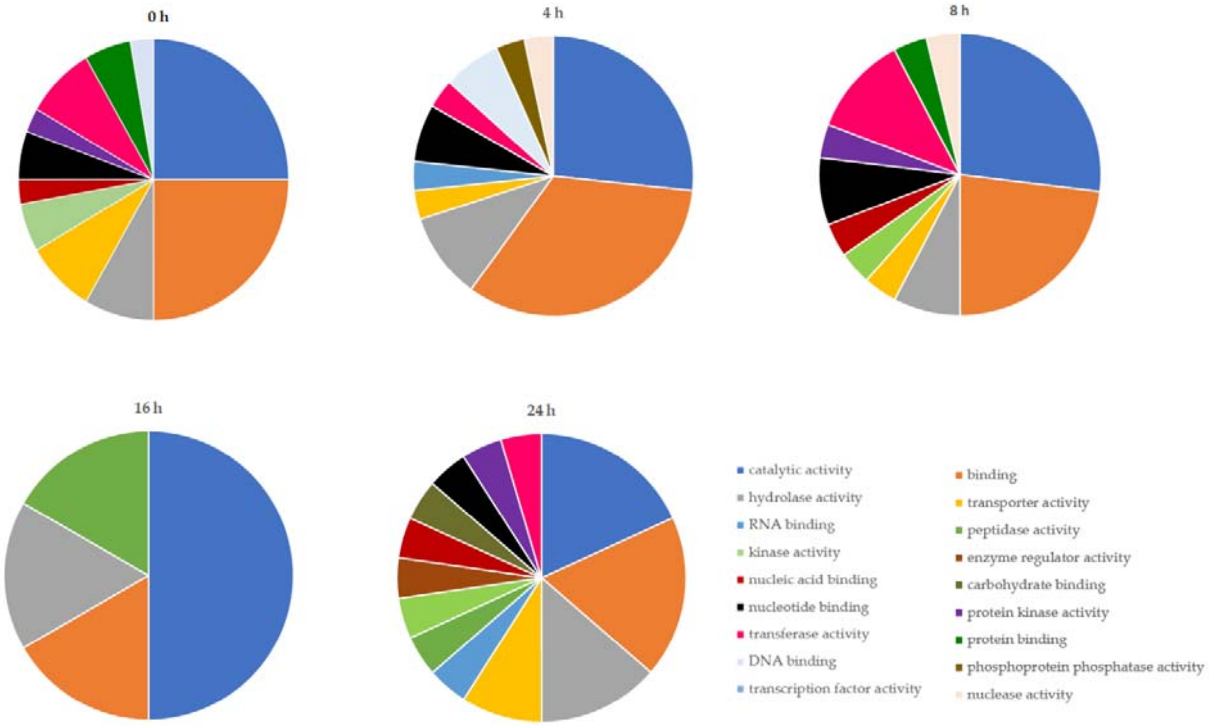

Supplementary Figure S2. Pie chart showing gene ontology (GO) classification of *Cryptococcus neoformans* responses to r-javanicin in each time point (0-24 h). Known proteins with different functions are indicated such as catalytic activity, hydrolase activity, transporter activity, RNA binding activity, etc.

Supplementary Table S1. The identified proteins of r-javanicin treated with *C. neoformans* at various time of incubations.

**Entry**	**Protein name**	**Gene ortholog (Molecular function)**
**a) Unique identified protein of *C. neoformans* treated with javanicin at 0 h (22)**		
1) A0A0S2LIY7	V-SNARE coiled-coil homology domain-containing protein	SNAP receptor activity [GO:0005484]; syntaxin binding [GO:0019905]
2) A0A225YFS0	Serine/threonine-protein kinase TTK/MPS1	ATP binding [GO:0005524]; protein serine/threonine/tyrosine kinase activity [GO:0004712]
3) A0A225YCL9	MAGE domain-containing protein	
4) A0A225YRK5	Sulfhydryl oxidase (EC 1.8.3.2)	flavin-linked sulfhydryl oxidase activity [GO:0016971]
5) A0A225YN69	Monosaccharide transporter	transmembrane transporter activity [GO:0022857]
6) A0A225X9Y7	Arf/Sar family, other	GTP binding [GO:0005525]
7) A0A225YVV9	Phytanoyl-CoA dioxygenase	dioxygenase activity [GO:0051213]
8) 0A225XLK9	Fe-S cluster assembly protein DRE2 (Anamorsin homolog)	2 iron, 2 sulfur cluster binding [GO:0051537]; 4 iron, 4 sulfur cluster binding [GO:0051539]; electron transfer activity [GO:0009055]; metal ion binding [GO:0046872]
9) A0A225XWE1	V-type ATPase, C subunit	proton transmembrane transporter activity [GO:0015078]
10) A0A225Z2A1	Vacuole morphology and inheritance protein 14	
11) A0A225XZ23	ATP-dependent bile acid transporter	ATPase activity [GO:0016887]; ATPase-coupled transmembrane transporter activity [GO:0042626]; ATP binding [GO:0005524]
12) A0A225Y2V2	E3 ubiquitin-protein ligase NRDP1	zinc ion binding [GO:0008270]
13) A0A0S2LIR3	Topoisomerase I damage affected protein 2	
14) A0A225Y9A9	DAGKc domain-containing protein	NAD+ kinase activity [GO:0003951]
15) A0A225Y689	HMG box factor, other	DNA binding [GO:0003677]
16) A0A225YL93	Glucoamylase	
17) A0A225Y170	Ferric-chelate reductase	oxidoreductase activity [GO:0016491]
18) A0A225XU63	Dolichyl-diphosphooligosaccharide-protein glycosyltransferase subunit WBP1	transferase activity [GO:0016740]
19) A0A225YI63	Antiphagocytic protein 1	
20) A0A225ZA98	SET domain-containing protein 6	
21) A0A225YZ51	Spore wall assembly-associated protein	
22) A0A225XEQ7	DNA dependent ATPase	hydrolase activity [GO:0016787]

Supplementary Table S1. (Continued)

Entry	Protein name	Gene ortholog (Molecular function)
**b) Unique identified protein of *C. neoformans* treated with javanicin at 4 h (15)**		
1) A0A225Z5T8	Protein phosphatase (EC 3.1.3.16)	metal ion binding [GO:0046872]; phosphoprotein phosphatase activity [GO:0004721]
2) A0A1Y0JXL3	Glyceraldehyde-3-phosphate dehydrogenase (Fragment)	oxidoreductase activity, acting on the aldehyde or oxo group of donors, NAD or NADP as acceptor [GO:0016620]
3) A0A225YTG2	H/ACA ribonucleoprotein complex non-core subunit NAF1	RNA binding [GO:0003723]
1) A0A225YJ92	Oxysterol-binding protein	
2) A0A225XTP6	Pyruvate decarboxylase	carboxy-lyase activity [GO:0016831]; magnesium ion binding [GO:0000287]; thiamine pyrophosphate binding [GO:0030976]
3) A0A225YP79	Niemann-Pick C1 protein	
4) A0A225YIU4	Hydrolase	hydrolase activity, acting on glycosyl bonds [GO:0016798]
5) A0A225Y9Q8	3-hydroxybutyryl-CoA dehydrogenase	3-hydroxyacyl-CoA dehydrogenase activity [GO:0003857]; NAD+ binding [GO:0070403]
6) A0A225XJE7	Protein KTI12	ATP binding [GO:0005524]
7) A0A225X7V2	Fungal_trans domain-containing protein	DNA binding [GO:0003677]; zinc ion binding [GO:0008270]
11) A0A225YAJ2	Forkhead transcription factor 3	DNA-binding transcription factor activity [GO:0003700]; sequence-specific DNA binding [GO:0043565]
12) A0A225XUT0	Dolichyl-diphosphooligosaccharide--protein glycosyltransferase subunit 1	transferase activity [GO:0016740]
13) A0A225XDI0	Hexose transporter	transmembrane transporter activity [GO:0022857]
14) A0A225Y9V2	tRNA-specific adenosine deaminase 1	adenosine deaminase activity [GO:0004000]; RNA binding [GO:0003723]
15) A0A225Y7C6	Inositol oxygenase (EC 1.13.99.1) (Myo-inositol oxygenase)	inositol oxygenase activity [GO:0050113]; iron ion binding [GO:0005506]

Supplementary Table S1. (Continued)

Entry	Protein names	Gene ontology (molecular function)
**c) Unique identified protein of *C. neoformans* treated with javanicin at 8 h (22)**		
1) A0A225YK20	Pre-mRNA-splicing helicase BRR2	ATP binding [GO:0005524]; helicase activity [GO:0004386]; nucleic acid binding [GO:0003676]
2) A0A225YAB0	UDP-glucose,sterol transferase	transferase activity [GO:0016740]
3) A0A225XF56	Meiosis induction protein kinase IME2/SME1	ATP binding [GO:0005524]; protein kinase activity [GO:0004672]
4) A0A225Z1C7	BTP domain-containing protein	protein heterodimerization activity [GO:0046982]
5) A0A225Y8Z0	N(6)-L-threonylcarbamoyladenine synthase (EC 2.3.1.234)	metal ion binding [GO:0046872]; N(6)-L-threonylcarbamoyladenine synthase activity [GO:0061711]
6) A0A225YGN4	Myosin heavy chain	
7) A0A225YLC6	Endonuclease (EC 3.1.30.-)	endonuclease activity [GO:0004519]; metal ion binding [GO:0046872]; nucleic acid binding [GO:0003676]
8) A0A225Y257	Alpha-ketoglutarate-dependent 2,4-dichlorophenoxyacetate dioxygenase	dioxygenase activity [GO:0051213]
9) A0A225YSN4	Transcription factor IIIB 90 kDa subunit	transmembrane transporter activity [GO:0022857]
10) A0A225XZ94	Glycosylphosphatidylinositol transamidase	
11) A0A225XR07	Blocked early in transport 1	
12) A0A225X9K2	Bis(5'-adenosyl)-triphosphatase	catalytic activity [GO:0003824]; nucleotide binding [GO:0000166]
13) A0A225X9V2	Cytochrome b5	heme binding [GO:0020037]; metal ion binding [GO:0046872]
14) A0A225YVY3	F-type H+-transporting ATPase subunit H	
15) A0A225Z4B6	Alpha,alpha-trehalose-phosphate synthase (UDP-forming)	catalytic activity [GO:0003824]
16) A0A225Z3I1	Structural maintenance of chromosomes protein 5	ATP binding [GO:0005524]
17) A0A0S2M5F3	DUF1746 domain-containing protein	
18) A0A225Z4A2	Amine oxidase	
19) A0A225XNZ4	FSH1 domain-containing protein	
20) A0A225YF70	Dolichyl-diphosphooligosaccharide-protein glycosyltransferase subunit OST5	
21) A0A225YMG3	Anaphase-promoting complex subunit 1	
22) A0A225XDB0	ABC transporter PMR5 (Fragment)	

Supplementary Table S1. (Continued)

Entry	Protein names	Gene ontology (molecular function)
**d) Unique identified protein of *C. neoformans* treated with javanicin at 16 h (12)**		
1) A0A225X9X4	L-lactate dehydrogenase (Cytochrome)	oxidoreductase activity [GO:0016491]
2) A0A225XF18	DPBB_1 domain-containing protein	
3) A0A225XBU3	Pre-mRNA-processing protein 45	
4) A0A225YMV4	Carboxypeptidase A4	metallocarboxypeptidase activity [GO:0004181]; zinc ion binding [GO:0008270]
5) A0A225XV75	Enoyl reductase	oxidoreductase activity [GO:0016491]
6) A0A225Y8E4	COP9 signalosome complex subunit 4	
7) A0A225Y883	Vacuolar protein-sorting protein BRO1	
8) A0A225Y6M6	GRASP55_65 domain-containing protein	
9) A0A225YW31	t-SNARE coiled-coil homology domain-containing protein	
10) A0A225YU84	Nucleolar pre-ribosomal-associated protein 1	
11) A0A225XFP3	Tricarboxylate carrier	
12) A0A225XJG9	CMP/dCMP deaminase zinc-binding protein	catalytic activity [GO:0003824]

Entry	Protein names	Gene ontology (molecular function)
**e) Unique identified protein of *C. neoformans* treated with javanicin at 24 h (21)**		
1) A0A225Y6C0	Solute carrier family 39 (Zinc transporter), member 1/2/3	metal ion transmembrane transporter activity [GO:0046873]
2) A0A225XGB0	Protein VTS1	mRNA binding [GO:0003729]
3) A0A225YLA8	FIST domain-containing protein	
4) A0A225XT73	PUB domain-containing protein	
5) A0A225XA81	ATP-binding cassette, subfamily B (MDR/TAP), member 1	ATPase activity [GO:0016887]; ATP binding [GO:0005524]
6) A0A225XF82	TFIIIC_sub6 domain-containing protein	
7) A0A225YC15	Mediator of RNA polymerase II transcription subunit 4 (Mediator complex subunit 4)	transcription coregulator activity [GO:0003712]
8) A0A225Z1Y5	2Fe-2S ferredoxin-type domain-containing protein	2 iron, 2 sulfur cluster binding [GO:0051537]; electron transfer activity [GO:0009055]
9) A0A0S2LIC7	Expressed protein	
10) A0A225YIU1	Stromal membrane-associated protein	GTPase activator activity [GO:0005096]
11) A0A225XR37	Glicosidase	carbohydrate binding [GO:0030246]; hydrolase activity, hydrolyzing O-glycosyl compounds [GO:0004553]

Supplementary Table S1. (Continued)

Entry	Protein names	Gene ontology (molecular function)
**e) Continued; Unique identified protein of *C. neoformans* treated with javanicin at 24 h (21)**		
12) A0A225X952	Calpain-like protease palB/RIM13	calcium-dependent cysteine-type endopeptidase activity [GO:0004198]
13) A0A225YPA6	Protoplast regeneration and killer toxin resistance protein	
14) A0A225XQI4	Suppressor protein SPT23	
15) A0A225XGM5	DUF4604 domain-containing protein	
16) A0A225XB55	U3 small nucleolar RNA-associated protein 20	
17) A0A225YYR3	Phospholipid transporter	transmembrane transporter activity [GO:0022857]
18) A0A225Y1Q2	SCY1-like	ATP binding [GO:0005524]; protein kinase activity [GO:0004672]
19) A0A225XDE8	GST N-terminal domain-containing protein	
20) A0A225YIA2	Allergen	
21) A0A225XUJ9	MFS multidrug transporter	transmembrane transporter activity [GO:0022857]
